# Subchronic modulation of bitter taste receptors (TAS2R) by procyanidins. Unravelling the complex interplay between stimulation and expression

**DOI:** 10.1007/s13105-025-01122-6

**Published:** 2025-09-24

**Authors:** Florijan Jalsevac, Maria Descamps-Solà, Adrià Vilalta, Helena Segú, M. Teresa Blay, Raúl Beltrán-Debón, Esther Rodríguez-Gallego, Ximena Terra, Anna Ardévol, Montserrat Pinent

**Affiliations:** Universitat Rovira i Virgili, Departament de Bioquímica i Biotecnologia, MoBioFood Research Group, C/Marcel·lí Domingo n°1, Tarragona, 43007 Spain

**Keywords:** Bitter taste receptors, Intestine, Rat, Chronic treatment, Grape-seed procyanidins, Epicatechin

## Abstract

Mediated by the bitter taste receptors (TAS2R), the perception of bitter taste does not only involve the oral cavity but various physiological systems throughout the gastrointestinal tract. The relationship between stimulation and modulation is crucial for understanding the broader implications of bitter taste signalling in health and disease. In this study, we investigated how the expression of intestinal rat Tas2r (rTas2r) is affected by natural extracts containing bitter ligands, examined their association with obesity, and their effects on GLP-1 secretion. For this, we performed subchronic stimulations with a mixture of polyphenols and individual molecules in rats. Moreover, we also examined how the individual bitter molecule (epicatechin) affects the secretory profile of intestinal enteroendocrine cells. Treating rats with procyanidins up-regulated rTas2r in all the segments of the gastrointestinal tract, with the most changes observed in the duodenum and ascending colon. Epicatechin, one of the main components of the previously used extract, had a much more specific effect, as we observed mostly changes in the jejunum, where rTas2137, -139, -143 and -144 were up-regulated. In Hutu-80 cells, epicatechin downregulated TAS2R14 after 24 hours, which limited GLP-1 secretion after acute peptone stimulation. Our results support a network effect in the role of the bitter taste receptors along the intestinal areas that must be considered to address the work with bitter agonists.

**Supplementary Information:**

The online version contains supplementary material available at 10.1007/s13105-025-01122-6.

## Key Points

- Bitter compounds influence the expression profile of intestinal receptors.
- Changes of TAS2R profile influence the functionality of enteroendocrine cells.

**Supplementary Information:**

The online version contains supplementary material available at 10.1007/s13105-025-01122-6.

## Introduction

The gustatory system plays a pivotal role in shaping dietary preferences and influencing overall health. Mediated by the bitter taste receptor (TAS2R) family of G protein-coupled receptors, bitter taste perception involves not just the oral cavity but various physiological systems throughout the gastrointestinal tract (GIT) [1–3]. These receptors can be found in the enteroendocrine cells of the gastrointestinal wall [4, 5]. Through these hormones, the whole cascade of metabolism, metabolic reactions, hormonal regulation, and regulation of satiety in the brain occurs [6, 7]. Understanding the intricate relationship between TAS2R stimulation and the modulation of TAS2R expression is essential if the broader implications of bitter taste signalling in health and disease are to be elucidated.

Grape-seed procyanidin extract (GSPE) is a complex mixture of several molecules that have proved to be agonists of several TAS2R [8, 9]. After intake, they undergo slight modifications in the upper GIT; in the intestine, mostly monomeric compounds are absorbed. However, a small amount of dimeric and trimeric forms is also absorbed, and all these molecules are then partially metabolised. The other bigger structures reach the lower GIT where, together with modified forms of the previously absorbed molecules secreted into the lumen of the intestine through the biliary secretions, are broken down and are modified by the microbiota [10]. Our group has proved that this extract has several health-promoting effects [11–15] (for example, in a context of diet-induced obesity, it is antiobesogenic) [16, 17]. Some of the antiobesogenic effects can be explained by how the extract affects food intake. When administered acutely, GSPE inhibits food intake in part through the induction of GLP-1 release [18]. We have shown that some of the flavanols in GSPE modulate enteroendocrine secretion through interactions with TAS2Rs [19]. However, the antiobesogenic effect of GSPE is not limited to food intake and, although there is little information available at present, TAS2R modulation may also play a role.

The agonism of some flavonoids on human TAS2R [8, 9, 20] (hTAS2R) and mouse Tas2r [21] (mTas2r) has been described in studies on the functional characterization of chemosensory receptors by heterologous expression in mammalian cell lines (HEK 293 T). Bioinformatic approaches have also been used to great effect to provide insight into the promiscuity and selectivity of various ligands binding to different TA2Rs [22–24]. In vivo studies of the administration of some mTas2r agonists have shown that activating these receptors has beneficial effects on health, such as enhanced glucose tolerance and insulin sensitivity, normalisation of lipids, and anti-inflammatory effects [6]. However, there is little information on the effects of the chronic stimulation of bitter taste receptors. Considering their relatively high promiscuity, the possibility of heterologous desensitisation should also be considered. Likewise, studies on human airway smooth muscle (HASM) cells have shown that TAS2R14 undergoes rapid agonist-promoted desensitization and can experience up to a 50% loss of function following repeated stimulation with agonist [25]. All the tested agonists, except for one, induced subsequent TAS2R14 internalization, as demonstrated by confocal microscopy showing receptor trafficking to early and late endosomes, which led to receptor down-regulation [25].

In this study, we hypothesise that some of the health effects of GSPE against diet-induced obesity can be mediated by their effects as ligands of bitter taste receptors. To address it, we investigated the in vivo response to subchronic GSPE stimulation on the most highly expressed rat Tas2r (rTas2r) in various intestinal regions [3], and examined the role played by rTas2r in the effects of GSPE under conditions of obesity. We aimed to elucidate the impact of subchronic epicatechin treatment on TAS2R expression and of gene expression alterations on the sensitivity of these receptors to stimulation.

## Materials and methods

### Animal experiments

#### GSPE treated cafeteria animals

The grape-seed proanthocyanidin extract (GSPE) was provided by Les Dérivés Résiniques et Terpéniques (Dax, France). According to the manufacturer, the composition of the GSPE used in this study (batch number 124029) includes monomers of flavan-3-ols (21.3%), dimers (17.4%), trimers (16.3%), tetramers (13.3%) and oligomers (5–13 units; 31.7%) [26]. A detailed analysis of composition ws provided by Margalef et a [26, 27].

**Fig. 1:**
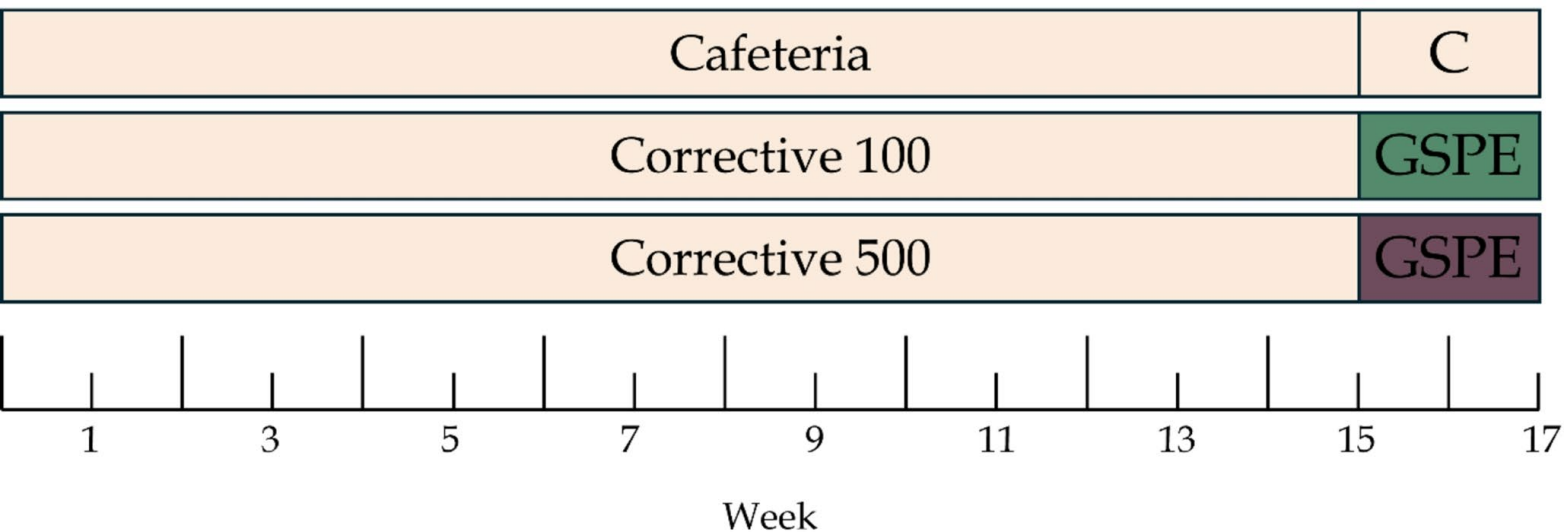
Schematic diagram of the experimental design with a cafeteria diet

Thirty female Wistar rats, each weighing between 240-270 g and seven weeks of age, were housed individually at a room temperature of 22 °C with a standard 12-h light-dark cycle. During a week of adaptation to the environment, the rats were fed ad libitum with a standard chow diet (Panlab 04, Barcelona, Spain) and tap water. Then, the rats were randomly distributed into three experimental groups (n = 10/group) all of which were fed with a cafeteria diet. The study design is summarized in Fig. 1 and has been described in depth elsewhere [26]. The three experimental groups were distributed as follows: CAFETERIA (CAF): fed with a cafeteria diet; CORRECTIVE 500 (500): fed with a cafeteria diet and given a corrective dose of 500 mg GSPE/kg of body weight (BW) (Human equivalent dose (HED): 81 mg/kg) for the last fifteen days of treatment; CORRECTIVE 100 (100), fed with cafeteria diet and given a corrective dose of 100 mg GSPE/kg (HED: 16.2 mg/kg) for the last fifteen days of treatment. The ”corrective” term for this diet is used to describe the fact that the animals have already been on the CAF diet and have developed some metabolic disruptions. We employed this study design to investigate whether these treatments can reverse some of the metabolic disruptions towards a healthier state. Both corrective GSPE treatments were given by oral gavage daily, one hour before the lights went off [28]. The control cafeteria group received a dose of tap water as a vehicle [26].

The cafeteria diet was administered every day for 17 weeks. It included bacon, biscuits with pâté, carrots, muffins, sausages, and sugared milk (the composition of the cafeteria diet can be found in the Table 1). Alongside of the cafeteria diet, the animals also had access to the standard chow diet.

**Table 1:** The composition of the cafeteria diet that was available to the animals

Component Offered	kJ/g	% Carbohydrate (g)	% Protein (g)	% Lipid (g)	% Fiber (g)
Bacon	14.43	1.0	14.9	31.7	0.0
Sausages	8.36	8.0	14.0	18.0	0.0
Paté	6.57	0.7	8.5	11.0	0.0
Biscuits	18.4	22.0	6.6	10.3	2.0
Muffins	18.8	30.0	4.1	23.1	1.7
Carrot	1.66	0.7	0.1	0.0	2.6
Milk	2.74	4.7	3.1	3.8	0.0
Sugar	16.73	100.0	0.0	0.0	0.0

At the conclusion of the study, the animals underwent a fasting period of 1–4 h before being anesthetized with sodium pentobarbital (70 mg/kg BW; Fagron Iberica, Barcelona, Spain) and subsequently exsanguinated from the abdominal aorta. Blood samples were obtained using heparin (Deltalab, Barcelona, Spain) as an anticoagulant. Then plasma was obtained through centrifugation and stored at −80 °C. Finally, the tissues were rapidly collected and stored at −80 °C. All procedures and the experimental design were approved by the Experimental Animal Ethics Committee of the Universitat Rovira i Virgili (code: 0152 S/4655/2015).

#### Epicatechin-treated animals

For this part of the study, we used eighteen 8-week-old female Wistar rats (Envigo, Barcelona, Spain). Upon arrival, rats were housed in pairs and underwent an adaptation period of 1 week, after which they were separated and again left to acclimatise for 1 week. All animals were kept under the same conditions, at a room temperature of 23 °C, with a standard 12-h light-dark cycle (lights on at 6:00) with ventilation. Animals had ad libitum access to tap water and a standard chow diet (2014 Teklad Global 14% protein rodent maintenance diet; Envigo, Barcelona, Spain). Afterwards the animals were distributed into two groups: Control, and Treatment. On the day of the treatment, rats were fasted, starting at 14:00, and the treatment was performed at 17:00, one hour before the lights were turned off. The treatment consisted of a dose of 400 mg/kg BW of epicatechin (HED: 64.8 mg/kg) (Cat. No.: E1753, Sigma-Aldrich, Madrid, Spain) dissolved in water. The treatment was performed by oral gavage. The control group received water by the same method. The food was introduced at 18:00. Next day, the food intake was measured at 14:00, giving a food intake of 20 h. This treatment was then repeated for eight more consecutive days, to give a total of nine days of treatment, while the food intake measurement was collected for the first 8 days of the treatment period. On the last day of treatment, the rats were fasted, starting at 23:00, and euthanized by decapitation next morning. The intestine and other tissues were carefully removed, measured, and weighed. Different intestinal segments were excised from the duodenum, the jejunum, the ileum, and the proximal colon. The entire intestinal tube of each segment was immediately frozen in liquid nitrogen and stored for subsequent analysis. All procedures and the experimental design were approved by the Experimental Animal Ethics Committee of Generalitat de Catalunya (authorization number 11700).

### Cellular assays

The enteroendocrine human HuTu-80 (ATCC, HTB-40) cell line was provided by LGCgroup (Barcelona, Spain). The cells were grown in culture flasks (Greiner Bio-One, Frickenhausen, Germany) at 37 °C in an atmosphere of 5% CO2 and the medium was changed every 2–3 days. The growth medium consisted of EMEM (Cat. No.: 30-2003, ATCC, Manassas, USA) supplemented with 10% v/v heat-inactivated foetal bovine serum (Cat. No.: 12103 C, Sigma-Aldrich, Madrid, Spain) and 100 U/mL penicillin-streptomycin mixture (Cat. No.: DE17-602E, Lonza Bioscience, Basel, Switzerland). When confluence reached roughly 80%, the cells were harvested by treatment with 0.25% Trypsin 1 mol/L EDTA (Cat. No.: T3924, Sigma-Aldrich, Madrid, Spain) for 5 min, and then split and sub-cultured in fresh medium.

#### Basal gene expression analysis

For this experiment, passages 13–17 were used. HuTu-80 cells (200,000 cells/mL, 1 mL per well) were seeded into individual culture plates (Greiner Bio-One, Frickenhausen, Germany). After 48 h, the cells were washed with cold PBS, and then the lysis buffer provided with the RNA extraction kit (RNeasy Plus Mini Kit; Cat. No.: 74134, Qiagen, Hilden, Germany) was added. The dishes were then frozen at −80 °C, until the RNA was extracted.

#### Epicatechin chronic treatment

##### Gene expression analysis

For this experiment, passages 10–12 were used. HuTu-80 cells (200,000 cells/mL, 1 mL per well) were seeded into 12-well plates. After 72 h, the cells were washed with PBS and treated for 24 h with epicatechin at either 10 or 50 µM. The stock solutions of the treatments were prepared in DMSO (Cat. No.: SU01581000, Scharlab, S.L., Barcelona, Spain) and then diluted to final working concentration in the complete growth medium. The final DMSO concentration was 0.05%. The control group was given fresh growth medium with added DMSO at the same concentration as the treatments (0.05%). The next day, the cells were washed with cold PBS, and then the lysis buffer provided with the above-mentioned RNA extraction was added. The plates were then frozen at −80 °C, until the RNA was extracted.

##### Enterohormone secretion analysis

For this experiment, passages 10–12 were used. HuTu-80 cells (200,000 cells/mL, 1 mL per well) were seeded into 12-well plates and treated with either 10 or 50 µM epicatechin for 24 h as described above. The next day, the medium was aspirated, and the cells were washed with PBS and treated for 2 h with peptone (5 mg of protein/mL) (Cat. No.: 70175, Sigma-Aldrich, Madrid, Spain). This treatment was prepared in glucose-free Krebs–Ringer buffer (KRB) (120 mM NaCl, 5 mM KCl, 2 mM CaCl2, 1 mM MgCl2, 22 mM NaHCO3). The control group received KRB, supplemented with the same final concentration of DMSO. After the final treatment, the mediums were collected and stored at −80 °C for subsequent analysis. Total GLP-1 secreted to the media was quantified using the commercial ELISA kit for GLP-1 (Cat No.: EZGLPT1-36k, Millipore, Madrid, Spain).

The cells were then washed with PBS, lysed with HEPES buffer supplemented with Triton^®^ X-100 (0.1%, Cat. No.: T8787, Sigma-Aldrich, Madrid, Spain) and stored at −80 °C. Total protein content was determined with a bicinchoninic acid (BCA) kit (Pierce, Thermo Fisher Scientific) and cytotoxicity in the cells was quantified with a lactate dehydrogenase assay (LDH) (QCA, Tarragona, Spain) as described elsewhere [29]. 

### Gene expression analysis

RNA from 1 cm at the beginning (or the middle in the jejunum) of each segment of the whole intestinal tissue, was extracted using Trizol (Cat. No.: 15596018; Invitrogen, USA) and trichloromethane-ethanol (Cat. No.:141252; Panreac, Barcelona, Spain). All the samples underwent the same procedure. Frozen samples were disrupted and homogenised using a TissueLyser LT small bead mill (Qiagen, Hilden, Germany).

RNA was obtained from the cell experiments using RNeasy plus mini kit. The procedure was performed by strictly following the instructions provided by the kit manufacturer.

The extracted RNA was evaluated for quality and purity using a NanoDrop^®^ ND-1000 spectrophotometer (Fisher Scientific, Madrid, Spain), and then stored at −80 °C. cDNA was obtained using the High-Capacity cDNA Reverse Transcription Kit (Cat. No: 4368814, Fisher Scientific, Madrid, Spain).

Quantitative PCR amplification was performed using specific TaqMan probes (Applied Biosystems, Waltham, USA) for all genes (40 ng/µL of cDNA) (found in supplementary Table 1). The relative expression of each gene was compared with that of the control group using the 2–∆∆Ct method. For the samples obtained from animals, Ppia was the reference gene, while for the cells, it was RPS9. Each figure indicates the analysis performed.

### Statistical analysis

All our results are expressed as mean ± standard error of the mean (SEM). P-values < 0.05 were considered statistically significant. For parametric data, we used Student’s T test to compare two groups or one-way ANOVA with the Bonferroni post hoc test to compare multiple groups. For non-parametric data, we worked with the Mann–Whitney U test to compare two groups, or the Kruskal–Wallis test by ranks with Dunn’s multiple comparison post hoc test to compare multiple groups. All calculations were performed using Lumivero XLSTAT 2023.1.5 software (Addinsoft, New York, NY, USA).

#### Integrative analysis and variable selection

The whole process of data processing, integration, variable selection, and statistical analysis outlined in this section was executed using RStudio version 2023.06.16 Build 446 (2009–2023 Posit Software, PBC).

The data on the parameters (biochemical, inflammation, intestinal permeability, enterohormones, and morphometrical) were collected, analysed and published elsewhere for various research purposes [11–14, 26, 30, 31]. All the initial data (35 bitter taste receptor gene expression results and 67 other parameters; expression of bitter taste receptors are presented in this paper, the complete list of the rest of parameters and where they have been published can be found in Supplementary Table 5) was subjected to pre-processing, including median imputation for missing values and the removal of redundant variables to make the data simpler and more straightforward. Subsequently, the data was adjusted and scaled using the ‘ScaleData’ function. Additionally, the ‘RunPCA’ function was used to help us to see if the samples grouped together or if any animals stood out and needed to be excluded in the next steps.

Once the data was clean, we used three machine learning methods – Elastic Net, Random Forest (RF), and PLS-DA, to look more closely at the variables in our multivariate approach throughout the selection process. We decided to use 3 different methods due to the robustness of this approach, as each method offered a different advantage. Elastic Net is particularly useful for managing datasets with numerous features, as it can effectively select the important variables while also handling multicollinearity. RF, a robust and adaptable algorithm belonging to the family of tree-based models, is suitable for both categorization and prediction tasks, specifically for highly dimensional datasets and capturing complex and non-linear relations between the parameters. PLS-DA is a versatile statistical method that excels at categorizing groups in complex datasets, so it is well-suited to our goal of distinguishing various experimental groups from the variables analysed, as it is a useful method to lower the dimensionality of the dataset, as well as being able to focus and enhance group separation. The parameters for each of the methods were optimised using 100 times repeated 5-fold cross-validation. To avoid overfitting, we used the glmnet R package and followed a rule that adds a little flexibility.

In this case, we applied these machine-learning algorithms to compare the cafeteria group first with the corrective 500 treatment and then with the corrective 100 treatment.

Each method produced scores reflecting the importance of variables in distinguishing between groups. These scores were treated as individual values for each variable within each method. The sum of the values obtained from the three methods yielded the final value. This score acted as an indicator of the variables’ overall importance in the study context. Variables were selected and ranked based on consensus importance measures from elastic net, PLS-DA, and random forest models, without statistical significance testing, representing those with the highest consensus-based discriminative power across models. The variable with the highest score was identified as the most significant variable in the study. Using these results, we conducted an integrative analysis that ranks all variables in order of importance. Using this selection method, we were able to concentrate future analyses on those variables with the highest discriminatory power between groups.

## Results

### Subchronic treatment with GSPE up-regulates most rTas2r located in jejunum in cafeteria-obese female rats

In this study, we investigated the impact of two subchronic treatments of GSPE on the expression of rTas2r. Our aim was to elucidate the potential involvement of predominant intestinal taste receptors [3] in mediating the effects of GSPE as part of a diet-induced obesity paradigm. To do so, we used a well-established cafeteria diet-induced obesity animal model, which serves as a robust platform for exploring the underlying mechanisms of obesity pathology [12].

**Fig. 2:**
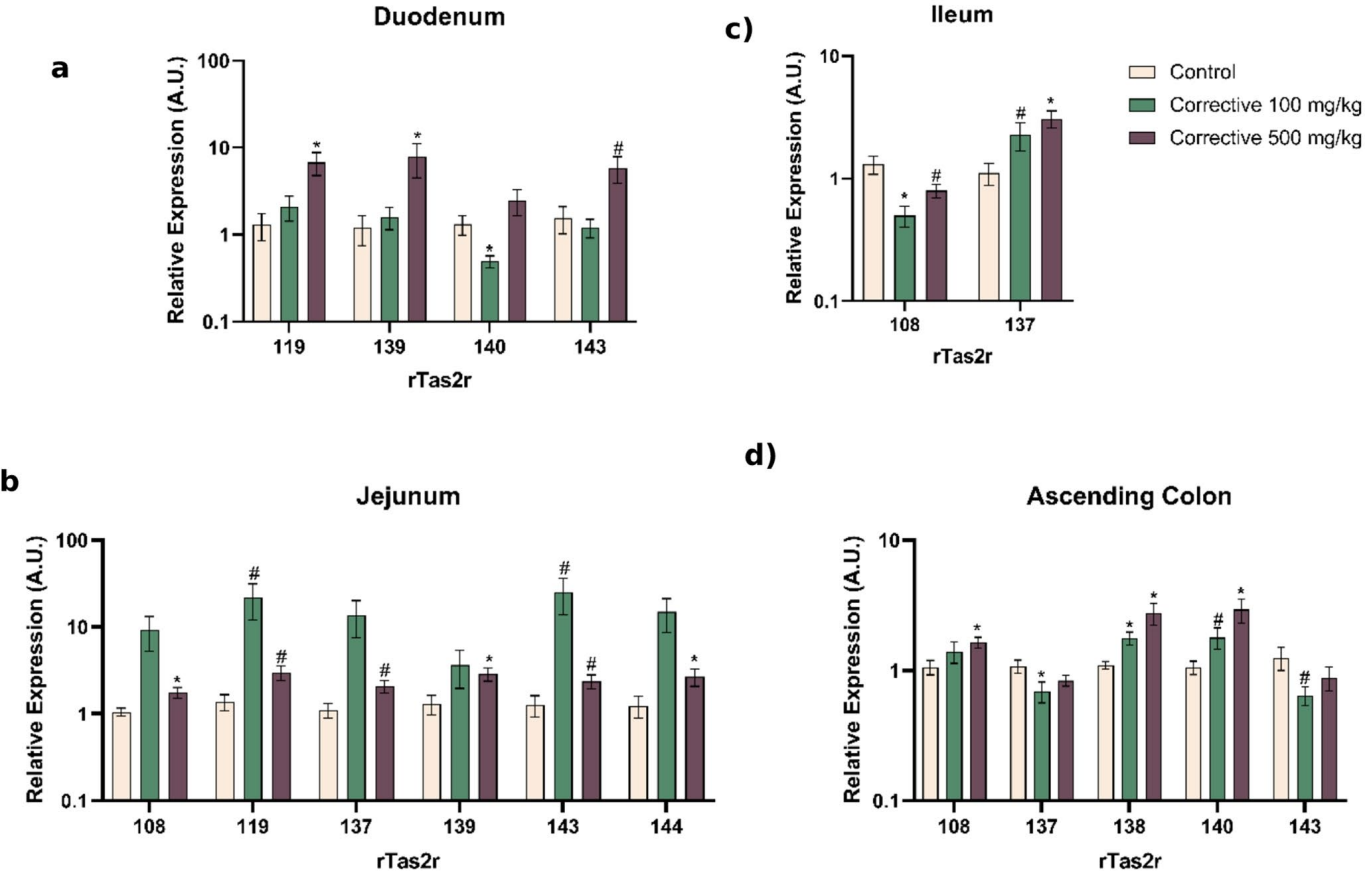
Relative expressions of rTas2s along the gastrointestinal tract (**A**) duodenum, (**B**) jejunum, (**C**) ileum, and (**D**) ascending colon. The Y-axis is a logarithmic scale for easier comparison. Values are mean ± SEM. * *p*-value < 0.05 compared with cafeteria group. # *p*-value < 0.1 compared with cafeteria group as control. *p*-value obtained using Kruskal-Wallis

We administered a dose of either 100 mg GSPE/kg BW or 500 mg GSPE/kg BW for 15 consecutive days to obese female rats before sacrifice [12–14, 31]. The highest dose induced greater changes in the rTas2r gene expression as can be observed in detail in Fig. 2 (rTas2r analysed without statistically significant effects can be found in Supplementary Table 2). We analysed the section between the duodenum and the ascending colon, and we found that the jejunum contained the highest number of GSPE-modified rTas2r. In duodenum, the 500 dose of GSPE significantly increased rTas2r119 and rTas2r139 and tended to increase the expression of rTas2r143. In ileum, only rTas2r137 was up-regulated by the highest dose, while − 108 tended to be down-regulated. In jejunum, the higher dose increased the gene expression of almost all of the analysed rTas2r, as only − 138 and − 140 did not show any statistically significant changes. Finally, in the ascending colon, the 500 dose increased the gene expressions of rTas2r108, −138 and − 140. And although GSPE 100 was barely effective in the small intestine, it was more effective at colonic site (Fig. 2), where it increased rTas2r138 and decreased rTas2r137 expressions. There was also a trend to increase rTas2r140 and decrease rTas2r143.

**Fig. 3:**
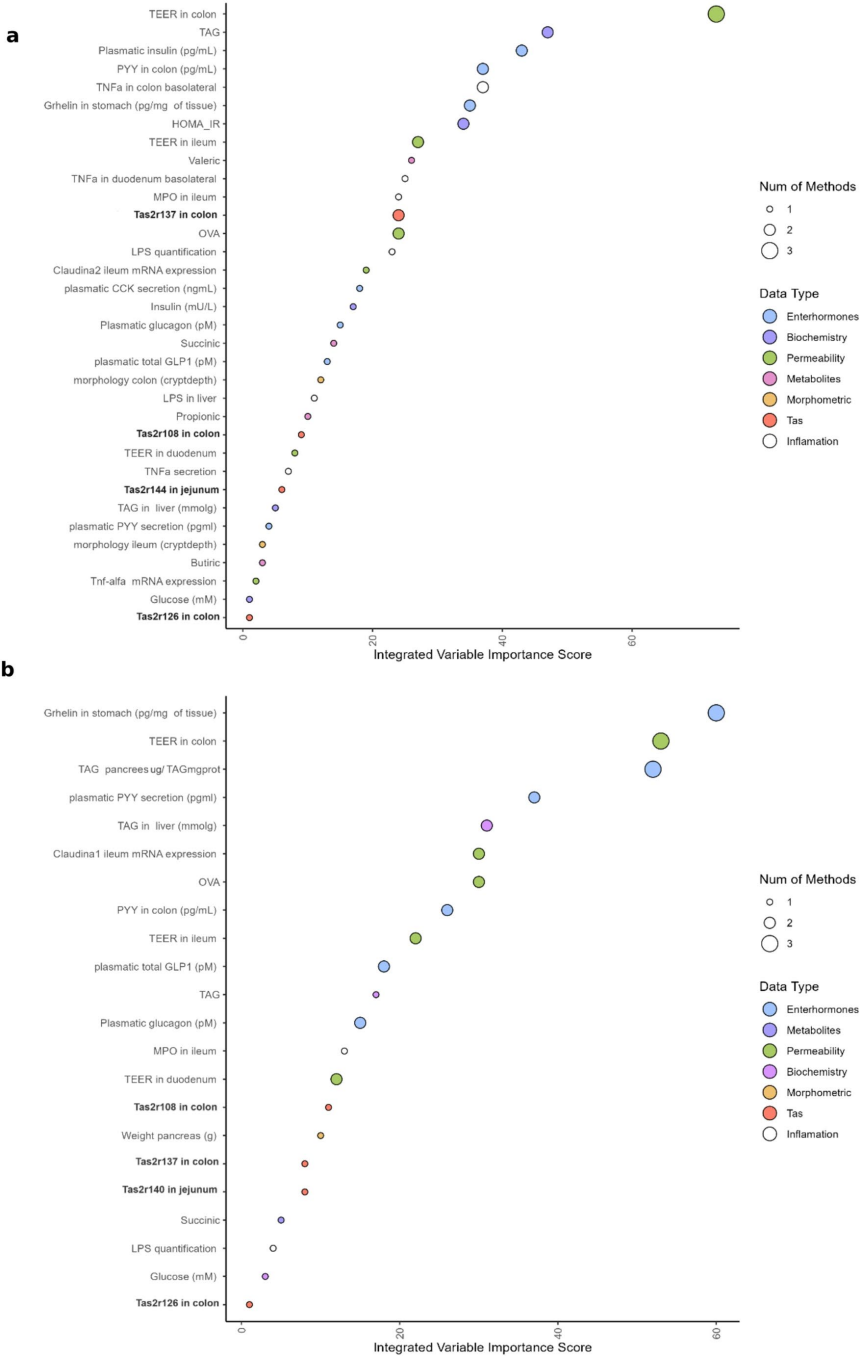
Principal variables distinguishing between the cafeteria group and corrective treatment groups. Integrate the analysis of selected variables using machine learning algorithms, ranking them to distinguish between (**A**) CAFETERIA and CORRECTIVE 500, and (**B**) CAFETERIA and CORRECTIVE 100, respectively

Elsewhere we have shown that these GSPE treatments are effective at improving the health status of these animals [12–14, 31]. To try to understand whether rTas2r are involved in the biological effect of GSPE, we integrated this information with the parameters analysed in those studies (biochemistry, inflammation, intestinal permeability, enterohormones, and morphometry) (Fig. 3). We used three machine-learning methods – Elastic Net, RF, and PLS-DA– to take a closer look at the variables in our multivariate approach throughout the selection process. We started with 102 parameters and after the three machine-learning methods had been implemented 34 were selected for the 500GSPE treatment and 22 for the 100GSPE treatment. The final score, obtained by aggregating the results from the three methods described in the Experimental section, indicates the variable’s overall importance. The variable, with the highest score is the most effective at discriminating between treatments.

From this comparison of the GSPE 500 group with the cafeteria group, we found that rTas2r137 expressed in ascending colon is the bitter taste receptor that best discriminates between obese rats and those treated with the 500 GSPE dose. It is positioned 13th among the parameters, indicating that it plays a major role in distinguishing between the two treatments. Of the 34 parameters considered (see Fig. 3A), we also observed that rTas2r108 from colon, −144, from jejunum and rTas126 from colon were in 24th, 27th and 34th position, respectively.

If we conduct the same analysis for the 100-dose group versus the cafeteria group, the first bitter taste receptor (rTas2r108 from colon) is the 15th of the 22 parameters characterised as important, (Fig. 3B). Additionally, Tas2r137 from colon, rTas2r140 from jejunum, and Tas2r126 from colon were in the 17th, 18th and 22nd positions, respectively.

### Subchronic treatment with epicatechin reproduces most of the GSPE effects on jejunal rTas2r

Given that GSPE is a complex mixture of various molecules, we opted to replicate a subchronic treatment using only one of its constituents: epicatechin. This compound is one of the most abundant monomeric constituents of GSPE and the primary product of the breakdown of GSPE’s oligomeric and polymeric structures [27]. It has also been identified as a specific agonist for bitter taste receptors. Specifically, in mice, at a concentration of 1 mM (threshold concentration) [21] epicatechin triggered activation of mTas2r126 and − 144. And according to Soares et al. [8], it was found to activate TAS2R4, −5, and − 39 when tested against human isoforms.

**Fig. 4:**
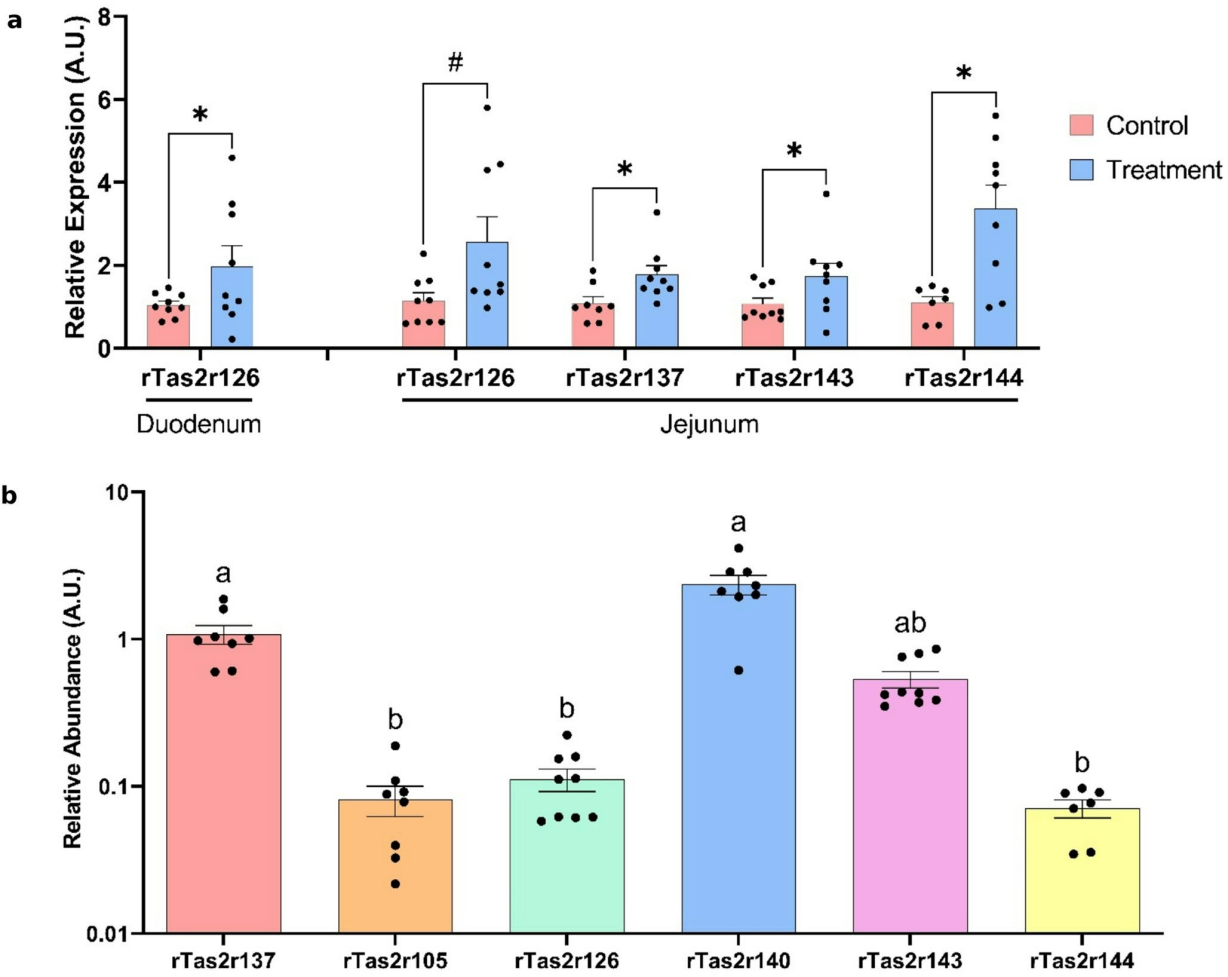
Relative expression of Tas2r in healthy rats. (**A**) Bitter taste receptors which have shown significant changes in their expression after a 9-day treatment with epicatechin 400 mg/kg BW (* *p* < 0.05; 0.05<#<0.1; either Student’s T-test or Mann–Whitney U test was used, depending on the distribution of the data; *n* = 7–9). (**B**) Relative abundances of rTas2r measured in jejunum, normalised to the rTas2r137. Y-axis is a logarithmic scale for ease of representation. The different letters signify significant differences (*p* < 0.05) between the groups (Kruskal–Wallis test by ranks with Dunn’s Multiple Comparison post hoc test; *n* = 7–9)

Healthy female Wistar rats were administered 400 mg of epicatechin/kg BW, after which we assessed alterations in gene expression within the duodenum, jejunum, ileum, and ascending colon. We specifically targeted receptors identified as isoforms (105, 126, and 144) [8, 14, 32], and some of those modified by GSPE in the previous study (137, 140, 143). The nine days of epicatechin treatment modified the gene expression of only some of the receptors assayed. Figure 4A shows the receptors which were statistically modified by the treatment, while those not affected are shown in supplementary Table 3. This treatment increased the expression of rTas2r126 in duodenum. As observed previously, the jejunum was again the intestinal segment with the highest number of receptors affected. Gene expression was increased by epicatechin treatment in rTas2r137, −143 and − 144 while in rTas2r126 this increase was only a trend. The ileum and ascending colon were not sensitive to this treatment. While different intestinal locations showed some discrepancies in the expression of rTas2rs, the most sensitive segment to the changes was jejunum. Thus, we decided to compare the relative abundances of different bitter taste receptors in this segment (Fig. 4B). The highest abundances were of rTas2r137 and − 140, followed by −143, while rTas2r144, −126 and − 105 showed lower abundances.

Given that the jejunum was highly sensitive to epicatechin treatment, we further examined the alterations in gene expression induced by epicatechin in an effort to reveal any similarities with the effects of GSPE. Subsequently, we completed our analysis on those rTas2r receptors that displayed the most significant modifications after GSPE treatment in the jejunum: rTAS2r108, −119, and − 139. We observed that rTas2r139 was increased by the treatment (Control: 1.18 ± 0.27; Epicatechin: 2.36 ± 0.41; *p* < 0.05 by Student’s T-test), while there were no significant effects on the expression of rTas2r108 (Control: 0.77 ± 0.11; Epicatechin: 1.19 ± 0.30; *p* > 0.1 by Student’s T-test). Given the significantly lower expression levels of rTas2r119 in these rats, this receptor cannot be considered for further analysis.

Finally, to investigate the effect of subchronic epicatechin treatment on healthy female rats in vivo, we measured two physiological parameters: body weight and daily food intake. After nine days of treatment, we observed that the epicatechin-treated group tended to gain less weight than the control group (% decrease vs. initial body weight: −1.02% ± 0.34% control vs. −1.75% ± 0.21% epicatechin; *p* = 0.093 by Student’s T-test). However, there was no statistically significant effect on the daily or accumulated food intake after eight days of treatment (20-hour food intake (kJ): 1452.3 ± 20. 2 control vs. 1440.5 ± 29.6 epicatechin; *p* = 0.747 by Student’s T-test).

### *Invivo* subchronic effects differ from in vitro effects in the Hutu-80 human cell line

**Fig. 5:**
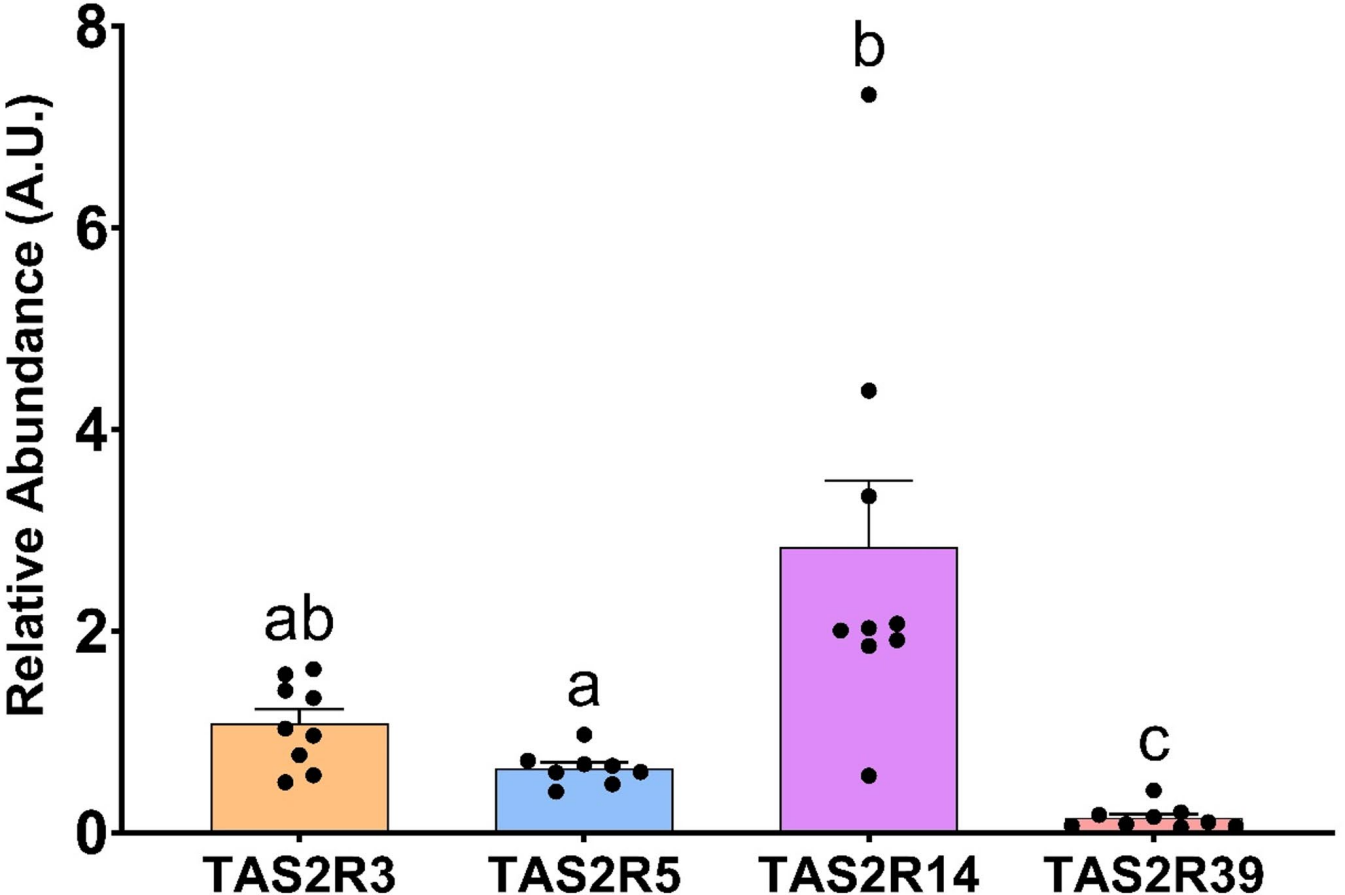
Relative abundances of four TAS2R in HuTu-80 cells, normalised to the TAS2R5. The groups that share the same letters are not significantly different (*p*-value: *p* < 0.05) as determined by the post hoc test (one-way ANOVA with the Bonferroni post hoc test; *n* = 8–9)

Previous in vivo investigations revealed an up-regulation of most bitter receptors after a 9-day treatment with bitter agonists. To establish a direct correlation between agonist stimulation, gene expression and biological activity, we transitioned to a cell-line approach. Specifically, we used the human Hutu-80 cell line, which had a distinct GLP-1 secretory capacity upon specific stimulation and expressed several intestinal TAS2R receptors [33]. To initially characterize our Hutu-80 cells, Fig. 5 depicts their higher abundance of hTAS2R14 (rat orthologue rTas2r140), moderate presence of hTAS2R3 (rTas2r137) and hTAS2R5 (no defined rat orthologue [32], and lower abundance of hTAS2R39 (rTas2r139).

After discounting that the treatments were in any way toxic (Supplementary Table 4), we treated the cells for 24 h with two doses of epicatechin (10 and 50 µM). We analysed the changes in the gene expression induced by these treatments. Table 2 shows that the lowest dose of epicatechin down-regulated the receptors that, according to the literature, do not bind epicatechin (hTAS2R3 and hTA2R14). Additionally, the two known receptors that do bind epicatechin, hTAS2R5 and hTAS2R39, displayed no differences in expression when treated.

**Table 2:** Changes in hTAS2R gene expression after chronic treatment with two doses of epicatechin in Hutu-80 cells. A total of 14–18 wells from at least 3 passages were used for each case. Student T-test. *: *p* < 0.05 vs. negative control; #: *p* < 0.1 vs. negative control; $: *p* < 0.1 vs. epicatechin 10

TAS2R	Treatment	Gene expression vs. negative control
hTAS2R3	Negative Control	1.082 ± 0.100
	Epicatechin 10	0.789 ± 0.083*
	Epicatechin 50	0.907 ± 0.097
hTAS2R5	Negative Control	1.061 ± 0.092
	Epicatechin 10	1.006 ± 0.071
	Epicatechin 50	1.081 ± 0.101
hTAS2R14	Negative Control	0.968 ± 0.104
	Epicatechin 10	0.758 ± 0.060#
	Epicatechin 50	0.928 ± 0.067$
hTAS2R39	Negative Control	1.249 ± 0.221
	Epicatechin 10	1.088 ± 0.150
	Epicatechin 50	0.975 ± 0.094

To test the importance of these down-regulations on the stimulation of GLP-1, after the chronic treatment we acutely stimulated the cells with peptone as a positive control for GLP-1 secretion [33]. The 24-hour treatment with 10 mM epicatechin reduced the basal, non-stimulated, GLP-1 secretion by 6% ± 2% (*p* < 0.05). However, this treatment also produced desensibilization to peptone stimulation, displaying only 69%± 4% of its ability to stimulate GLP-1 secretion without the chronic treatment. It has been reported that some peptides are ligands of hTAS2R14 [34]. Thus, our results suggest that the effect of epicatechin down-regulating hTAS2R14 expression (Table 2) may be an explanation for the lower stimulation shown by peptone [35]. Although further work is needed to conclude it.

## Discussion

Our objective was to investigate the relationship between the sustained stimulation of bitter taste receptors and their implications for metabolic health. We also sought to examine the specificity of this stimulation and its effects on metabolic outcomes. To do so, we worked with three experimental designs: a subchronic study with a mixture of several agonists of Tas2r in an obesity animal model, a subchronic study with a specific agonist (epicatechin) in a healthy animal, and 24-hour epicatechin administration to a human enteroendocrine cell line.

While in rats we can find approximately 36 different transcripts for Tas2rs, we focused our analysis on a handful of receptors which have been observed to be present in the intestines of the animals [3], in conjunction with the receptors that are more likely to be activated by the GSPE extract [9]. One characteristic of these receptors is that they require relatively high concentrations of ligands to be activated [9, 21]. This situation is more likely to happen in the lumen of the GIT than in inner body locations (liver, adipose tissue, brain…), since the Tas2Rs are located in the intestinal wall. In this way, it must be considered the low bioavailability of several of these ligands [10], favouring a higher concentration of them to be found in the GIT lumen [10]. These doses are far from toxic effects [36]. In both animal studies, after estimating the equivalent human doses, we identified amounts that are more likely to be achieved using concentrated extracts (93.31 mg Epicatechin/100 g) [37]. Although this study primarily adopts a nutraceutical approach, utilising doses unlikely to be attained through dietary intake alone—doses typically required to demonstrate a measurable biological effect—this does not preclude the potential for significant benefits from lower, diet-achievable levels, particularly when consumed consistently over an extended period. For example, regular consumption of epicatechin-rich foods like cocoa (31.22 mg/100 g) [37] or black soybeans (37.4 mg/100 g) [37] could help reach the local intestinal concentrations necessary to achieve the effects observed in the cell studies.

Following subchronic treatment with 500 GSPE, a complex mixture of TAS2R agonists, we observed a marked upregulation of rTas2r expression in the jejunum, particularly for subtypes rTas2r108, −119, −137, −139, −140 and − 144, suggesting activity of the raw-monomeric forms of GSPE in the upper GIT. These effects were dose-dependent and confined to regions where GSPE remains briefly before microbial metabolism occurs [38, 39]. In contrast, in the colon, where GSPE is retained longer and metabolized—both GSPE doses upregulated rTas2r138 and − 140, implicating these receptors in responses to GSPE-derived metabolites, despite limited data on their specific ligand interactions.

To explore this further, we tested epicatechin, a known GSPE component, in healthy rats. We defined a dosage to simulate all the monomeric epicatechin that can be derived from the higher dose of GSPE. It can be estimated from the GSPE epicatechin (the monomeric amount previously measured [27] plus epicatechin derived from the digestion of oligomeric and polymeric forms along the GIT. It induced upregulation of jejunal rTa2r137, −139, −143, and − 144, supporting the hypothesis that the epicatechin component in GSPE contributes to its activity via TAS2R engagement.

Data on chronic TAS2R agonist effects are limited. There are some studies on sweet taste receptors [42] and bitter compounds like ellagitannins [43] that suggests prolonged exposure can modulate receptor expression and intestinal physiology. Specifically, bitter agonists increased colonic expression of mTas2r subtypes, attenuated inflammation, and enhanced GLP-1 secretion—findings consistent with our observations of TAS2R gene upregulation and improved inflammatory profiles following GSPE treatment [12, 13].

To relate the effects on gene expression with the effects on metabolic health [12–14], we ran an integrative analysis that identified some of the bitter taste receptors that played a role in the beneficial effects of GSPE against obesity. We ran two integrative analyses, one for each GSPE dose, and although conducted separately, they consistently identified similar key parameters (TEER colon, ghrelin abundance in stomach, triglycerides in pancreas and PYY in colon). This reinforces the idea that these variables are effective at distinguishing between groups treated with GSPE and those exposed to a cafeteria diet. Since we analysed the same parameters for both doses, the differences between them could be related to the dose of GSPE. The bitter taste receptors modified by these treatments showed relatively low positions in these analyses, which identified three Tas2r as important in colon: Tas2r108, −126 and − 137. This suggests that these receptors are important for GSPE effects in the colon. The important Tas2r in the jejunal was different for the two doses, however. For 500 GSPE, it was Tas2r144 while for 100 GSPE it was Tas2r140. Very few studies have been done on rats and bitter taste receptors, but the limited information available can help to understand our results. Only mTas2r108 has clearly been shown to be a good target for counteracting metabolic derangements [6] and TAS2R38 seems to be important for the stimulation of GLP-1 secretion in enteroendocrine L-cells [44].

As all the previous analyses and published works unexpectedly showed an up-regulation of bitter taste receptors after chronic stimulation, we were interested in gaining greater insight into the relation between the defined agonisms and gene expression changes. In the study with epicatechin in vivo, the two receptors that bind to epicatechin were up-regulated. rTas2r126 showed an up-regulation in the duodenum and jejunum, and rTas2r144 in the jejunum, suggesting that they were more sensitive to stimulation by their defined ligand in the upper GIT where epicatechin has not yet been modified [10, 45, 46]. We also found that several other rTas2r which have not been defined as targets for epicatechin had been up-regulated [21]. But this is not a common mechanism after the chronic stimulation of receptors.

The cell-line study showed that 24 h of simulated chronic epicatechin stimulation down-regulated receptors, as expected, but surprisingly did not modify the gene expression of the hTAS2R for which epicatechin has been shown to be an agonist. We found that epicatechin, an agonist of hTAS2R4, −5 and − 39, downregulates hTAS2R3 and − 14. And, in this case, down-regulation induced a lower stimulation by peptone suggesting heterologous desensitization. These effects have also been found by experiments conducted in human airway smooth muscle (HASM) cells, which have also shown that hTAS2R14 undergoes rapid agonist-promoted desensitization that can lead to a 50% loss of function (although some agonists caused minimal desensitization) [25]. These experiments also assayed long-term desensitization with HASM cells in real time. The cells were treated with vehicle or hTAS2R agonist for 18 h and then challenged with an additional higher final dose of agonist with an immediate readout of Ca^2+^ [35]. Molecular docking studies showed that deep within the binding pocket of the hTAS2R14 there were specific interactions between the agonists that induced desensitization and the one that did not [35]. The results we report here, however, show desensitization by compounds that are not defined ligands of hTAS2R3 and − 14. It can be explained as heterologous desensitization. This does not provide an explanation for our in vivo effects, but the complexity of the system can induce a myriad of mechanisms that produce the up-regulations. We cannot discard specific species differences between the two models assayed either, but the differences in the degree of the complexity of the systems (in vivo versus in vitro) is the most likely reason for both effects.

Our data indicate that activation of specific TAS2Rs by their selective ligands triggers a broader sensitisation response within the bitter taste receptor family. This is reflected not only in the upregulation of the receptors directly engaged by these ligands, but also in the increased expression of other TAS2Rs for which no established binding interaction exists. Such a response suggests a form of cross-sensitisation, ultimately leading to enhanced overall sensitivity to bitter compounds. This effect is interesting in animals subjected to a cafeteria-style diet, which suffer a global downregulation. Under these conditions, the observed increase in TAS2R responsiveness may represent an adaptive or compensatory mechanism that mitigates some of the deleterious consequences of the diet, potentially conferring a beneficial physiological effect.

However, given the currently limited understanding of the functional roles of individual TAS2Rs, it would be premature to ascribe improved physiological outcomes to specific receptor subtypes. Notably, our results clearly indicate that the effects of TAS2R agonists are segment-specific within the intestine. This observation opens the door for future investigations into the functional relationships between individual TAS2Rs and distinct intestinal regions. Among these, the duodenum and jejunum emerge as particularly relevant sites, potentially linked to the metabolic adaptations observed following bariatric procedures [47–52]. Such surgeries, typically employed in the management of morbid obesity, are associated with marked alterations in inflammatory and enteroendocrine profiles, reflecting enhanced gut-organ communication. The localisation of TAS2R-related effects within the upper gastrointestinal tract thus provides a compelling rationale for the development of targeted anti-obesity therapies acting via these receptors, potentially offering a non-surgical alternative to bariatric interventions.

Importantly, this study was conducted in female animals, thereby offering novel insights into potential sex-specific effects—an area that remains largely underexplored. To date, investigations into sex or gender differences in TAS2R function have largely centred on human polymorphisms, particularly in TAS2R38 [40, 41]. No previous studies, to our knowledge, have addressed the chronic or subchronic administration of bitter agonists with respect to sex-related differences in metabolic outcomes, or in their impact on TAS2R expression and distribution along the intestinal tract.

## Conclusions

In summary, subchronic bitter taste stimulation mainly induces an up-regulation of several bitter taste receptors in vivo in the upper GIT. Treatment with epicatechin up-regulated its specific rTas2r, but also some others, which suggests an increased sensitivity to stimulation. Working on the cell line, we also found heterologous desensitisation, reinforcing the idea that there is a “network effect” in the role of the bitter taste receptors in the upper GIT which should be considered when addressing effects of bitter agonists.

But some more work is needed to clearly consolidate these first evidence regarding the interplay between different bitter taste receptors. Until now, there is scarce knowledge regarding their extraoral roles. More research is needed to further describe the mechanisms responsible for the observed effect. All this knowledge will help to produce in the future a fine-tuned nutritional approach through the specific stimulation of some of the bitter taste receptors to modulate satiety and/or metabolic health.

## Supplementary Information

Below is the link to the electronic supplementary material.

Supplementary Material 1

## Data Availability

The data published in this article can be found at the CORA depository at the following link: https://dataverse.csuc.cat/dataset.xhtml?persistentId=doi:10.34810/data1783.
