# Supplementary material for: Subchronic modulation of bitter taste receptors (TAS2R) by procyanidins. Unravelling the complex interplay between stimulation and expression
Source: J Physiol Biochem. 2025 Sep 24;81(4):1321–34. doi: 10.1007/s13105-025-01122-6 (PMC12738599; doi:10.1007/s13105-025-01122-6)
Supplement: Supplementary file 1 — Supplementary Material 1 [file 13105_2025_1122_MOESM1_ESM.docx]

**Subchronic modulation of bitter taste receptors (TAS2R) by procyanidins Unravelling the complex interplay between stimulation and expression**

**Supplemental Data**

*Supplementary Table 1: Names and reference numbers of the TaqMan probes for rats and humans used in the gene expression analysis.*

| Rat | | Human | | |
| --- | --- | --- | --- | --- |
| Receptor | TaqMan Ref. Num. | Receptor | TaqMan Ref. Num. |  |
| *rTas2r105* | Rn00577003_s1 | *TAS2R3* | Hs00249942_s1 |  |
| *rTas2r108* | Rn02396427_s1 |  |  |  |
| *rTas2r119* | Rn00576950_s1 | *TAS2R5* | Hs01549633_s1 |  |
| *rTas2r126* | Rn00595098_s1 |  |  |  |
| *rTas2r137* | Rn01500928_s1 | *TAS2R14* | Hs00256800_s1 |  |
| *rTas2r139* | Rn04218919_s1 |  |  |  |
| *rTas2r140* | Rn01492598_s1 | *TAS2R39* | Hs00603443_s1 |  |
| *rTas2r143* | Rn02585801_s1 |  |  |  |
| *rTas2r144* | Rn02585844_s1 | *Rps9* | Hs02339424_g1 |  |
| *Ppia* | Rn00690933_m1 |  |  |  |

|  | Receptor | Control | | Corrective 100 | | Corrective 500 | |
| --- | --- | --- | --- | --- | --- | --- | --- |
|  |  | Relative Expression (A.U.) | N | Relative Expression (A.U.) | N | Relative Expression (A.U.) | N |
| Duodenum | *rTas2r108* | 1.648 (± 0.52) | 10 | 0.729 (± 0.11) | 9 | 2.080 (± 0.60) | 8 |
|  | *rTas2r137* | 1.300 (± 0.44) | 9 | 0.647 (± 0.12) | 9 | 2.385 (± 0.83) | 8 |
|  | *rTas2r144* | 1.521 (± 0.55) | 8 | 0.944 (± 0.23) | 9 | 4.453 (± 1.50) | 9 |
| Jejunum | *rTas2r119* | 1.377 (± 0.29) | 7 | 21.671 (± 9.61) | 7 | 2.975 (± 0.56) | 6 |
|  | *rTas2r137* | 1.105 (± 0.21) | 6 | 13.790 (± 6.32) | 7 | 2.081 (± 0.35) | 6 |
|  | *rTas2r138* | 1.338 (± 0.35) | 6 | 17.492 (± 7.02) | 7 | 2.105 (± 0.34) | 6 |
|  | *rTas2r144* | 1.243 (± 0.35) | 6 | 14.963 (± 6.26) | 7 | 2.691 (± 0.62) | 6 |
| Ileum | *rTas2r119* | 1.34 (± 0.30) | 3 | 0.035 (± 0.01) | 4 | 0.238 (± 0.01) | 6 |
|  | *rTas2r126* | 1.082 (± 0.17) | 4 | 0.935 (± 0.33) | 4 | 1.749 (± 0.40) | 7 |
|  | *rTas2r138* | 1.000 (± 0.11) | 3 | 0.108 (± 0.02) | 5 | 0.349 (± 0.11) | 7 |
|  | *rTas2r139* | 1.003 (± 0.15) | 4 | 0.055 (± 0.01) | 4 | 0.238 (± 0.10) | 6 |
|  | *rTas2r140* | 1.250 (± 0.12) | 4 | 1.864 (± 0.52) | 5 | 2.323 (± 0.62) | 7 |
|  | *rTas2r143* | 1.157(± 0.24) | 4 | 0.316 (± 0.06) | 4 | 0.994 (± 0.14) | 7 |
|  | *rTas2r144* | 1.085 (± 0.20 | 4 | 0.032 (± 0.01) | 4 | 0.198 (± 0.07) | 6 |
| Ascending Colon | *rTas2r119* | 1.117 (± 0.17) | 10 | 1.274 (± 0.25) | 8 | 1.623 (± 0.44) | 7 |
|  | *rTas2r126* | 1.210 (± 0.24) | 10 | 0.799 (± 0.10) | 10 | 1.279 (± 0.23) | 8 |
|  | *rTas2r139* | 1.161 (± 0.18) | 10 | 1.065 (± 0.08) | 8 | 1.173 (± 0.13) | 8 |
|  | *rTas2r144* | 1.218 (± 0.20) | 9 | 1.737 (± 0.34) | 8 | 1.567 (± 0.15) | 9 |

Supplementary Table 2: Relative expressions of individual bitter taste receptors in each individual segment of the intestine, where no statistical differences were observed between the control and GSPE treated groups. Data represented Average Relative Expression (normalised to the control group for each individual receptor) ± SEM.

Supplementary Table 3: Relative expressions of individual bitter taste receptors in each individual segment of the intestine, where no statistical differences were observed between the control and treated group. Data represented Average Relative Expression (normalised to the control group for each individual receptor) ± SEM.

|  | Receptor | Control | | Treatment | |
| --- | --- | --- | --- | --- | --- |
|  |  | Relative Expression (A.U.) | N | Relative Expression (A.U.) | N |
| Duodenum | *rTas2r105* | 1.201 (± 0.33) | 6 | 1.196 (± 0.32) | 6 |
|  | *rTas2r137* | 1.023 (± 0.10) | 9 | 1.153 (± 0.13) | 9 |
|  | *rTas2r140* | 1.207 (± 0.23) | 9 | 0.983 (± 0.22) | 8 |
|  | *rTas2r143* | 1.030 (± 0.08) | 8 | 1.494 (± 0.34) | 9 |
|  | *rTas2r144* | 1.182 (± 0.20) | 9 | 0.880 (± 0.16) | 8 |
| Jejunum | *rTas2r105* | 1.253 (± 0.29) | 8 | 1.574 (± 0.28) | 8 |
|  | *rTas2r140* | 1.118 (± 0.17) | 8 | 6.206 (± 2.66) | 9 |
| Ileum | *rTas2r105* | 1.525 (± 0.44) | 9 | 2.074 (± 0.48) | 9 |
|  | *rTas2r126* | 1.103 (± 0.18) | 9 | 1.601 (± 0.37) | 9 |
|  | *rTas2r137* | 1.239 (± 0.22) | 9 | 1.090 (± 0.11) | 9 |
|  | *rTas2r140* | 1.148 (± 0.20) | 8 | 1.827 (± 0.50) | 9 |
|  | *rTas2r143* | 1.170 (± 0.22) | 9 | 1.401 (± 0.24) | 9 |
|  | *rTas2r144* | 1.158 (± 0.23) | 9 | 1.166 (± 0.18) | 9 |
| Ascending Colon | *rTas2r105* | 1.188 (± 0.42) | 4 | 0.269 (± 0.11) | 4 |
|  | *rTas2r126* | 1.223 (± 0.22) | 8 | 1.065 (± 0.15) | 9 |
|  | *rTas2r137* | 1.104 (± 0.14) | 9 | 1.114 (± 0.12) | 9 |
|  | *rTas2r140* | 1.041 (± 0.11) | 7 | 1.377 (± 0.09) | 7 |
|  | *rTas2r143* | 1.080 (± 0.18) | 9 | 0.743 (± 0.12) | 9 |
|  | *rTas2r144* | 1.166 (± 0.19) | 9 | 0.902 (± 0.16) | 9 |

Supplementary Table 4: The percentage of toxicity ± SEM of different treatments of the 24-hour treatments of the HuTu-80 cells. This assay measures the activity of the enzyme Lactate dehydrogenase, which is rapidly secreted from the damaged or dead cells. The assay compares the activity in the cellular medium and in the lysate of the cell, and the ration of these 2 measurements is processed and the percentage of toxicity is obtained. No significant differences have been observed between the treatments.

|  | Toxicity % | N |
| --- | --- | --- |
| Control | 9.40 ± 0.43 | 6 |
| Epicatechin 10μM | 10.09 ± 0.27 | 6 |
| Epicatechin 50μM | 10.40 ± 0.30 | 6 |

*Supplementary Table 5: The table of the parameters (all except for the results of the bitter taste receptor gene analysis, which is described in this paper) which have been used for the multivariate analysis, and where they have been published or can be found.*

| Reference | Parameters | |
| --- | --- | --- |
| González-Quilen C, Gil-Cardoso K, Ginés I, Beltrán-Debón R, Pinent M, Ardévol A, Terra X, Blay MT. Grape-Seed Proanthocyanidins are Able to Reverse Intestinal Dysfunction and Metabolic Endotoxemia Induced by a Cafeteria Diet in Wistar Rats. Nutrients. 2019 Apr 29;11(5):979. | Adiposity | Non-Esterified Fatty Acids Levels in Plasma |
|  | Brown Adipose tissue Weight | Glucose Levels in Plasma |
|  | Liver weight | Insulin Levels in Plasma |
|  | Pancreas weight | HOMA-IR |
|  | Spleen weight | TNF-α Levels in Plasma |
|  | Thymus weight | LPS Levels in Plasma |
|  | Kidney weight | Myeloperoxidase activity in Ileum |
|  | Triacylglycerol Levels in Plasma | ROS in Ileum |
|  | Liver Triglycerides Levels in Plasma |  |
| Ginés I, Gil-Cardoso K, Terra X, Blay M, Pérez-Vendrell AM, Pinent M, Ardévol A. Grape Seed Proanthocyanidins Target the Enteroendocrine System in Cafeteria-Diet-Fed Rats. Mol Nutr Food Res. 2019 Jun;63(11):e1800912. | CCK Levels in Plasma | Ghrelin content per mg of stomach |
|  | PYY Levels in Plasma | PYY in Ileum (Ussing Chamber) |
|  | Active Ghrelin Levels in Plasma | PYY in Colon (Ussing Chamber) |
|  | GLP-1 Total Levels in Plasma | GLP-1 Active Levels in Plasma |
|  | Short-Chain Fatty Acids (formic, acetic, propionic, butyric, valeric, succinic) Levels in Plasma | |
| González-Quilen C, Gil-Cardoso K, Ginés I, Beltrán-Debón R, Pinent M, Ardévol A, Terra X, Blay MT. Grape-Seed Proanthocyanidins are Able to Reverse Intestinal Dysfunction and Metabolic Endotoxemia Induced by a Cafeteria Diet in Wistar Rats. Nutrients. 2019 Apr 29;11(5):979. | OVA Levels in Plasma | Occludin expression in Ileum |
|  | transepithelial electrical resistance in Ileum | Tight junction protein 1 expression in Ileum |
|  | transepithelial electrical resistance in Duodenum | Claudin 2 expression in Ileum |
|  | transepithelial electrical resistance in Colon | Claudin 3 expression in Ileum |
|  | Claudin 1 expression in Ileum |  |
| Parameters which have not been published yet, but will be deposited in the open assess repository CORA Research Data Repository (https://www.csuc.cat/en/serveis/cora-repositori-de-dades-de-recerca) | Small intestine length | Insulin content per mg of Pancreas |
|  | Large intestine length | CD36 expression in Ileum |
|  | Duodenum Weight | Scarb1 expression in Ileum |
|  | Ileum Crypt Depth | CD36 expression in Duodenum |
|  | Colon Crypt Depth | Scarb1 expression in Duodenum |
|  | Amylin Levels in Plasma | CD36 expression in Colon |
|  | Glucagon Levels in Plasma | Scarb1 expression in Colon |
|  | LPS levels in Liver | Cannabinoid receptor 1 expression in Colon |
|  | LPS levels in Mesenteric Adipose Tissue | Junctional adhesion molecule A expression in Ileum |
|  | TNF-α in Ileum (Ussing Chamber) | Lucifer Yellow Ileum |
|  | TNF-α in Duodenum (Ussing Chamber) | Lucifer Yellow Duodenum |
|  | TNF-α in Colon (Ussing Chamber) | Lucifer Yellow Colon |
|  | TAG content per mg of Pancreas |  |
